# Development of a standard operating procedure for investigational product rights of administration in clinical trials

**DOI:** 10.1017/cts.2025.10181

**Published:** 2025-10-23

**Authors:** Lauren Hill, Carolynn Thomas Jones

**Affiliations:** 1 Cincinnati Children’s Hospital Medical Center, Translational Pulmonary Science Center, Cincinnati, OH, USA; 2 The Ohio State University, College of Nursing and Clinical Translational Science Institute, Columbus, OH, USA

**Keywords:** Investigational new drug, investigational medicinal product, rights of medication administration, standard operating procedures, ICH E6(R3)

## Abstract

Medication errors in clinical care and in clinical research are preventable situations requiring quality improvement approaches to mitigate negative safety trends. The “Rights of Medication Administration” framework has existed in hospital and clinic settings for decades to aid clinicians with ensuring medication administration safety for patients. These quality measures such as expanded rights of medication administration, bar coding, and “time outs” have been employed to improve clinical patient safety. In clinical trials, drug accountability standard operating procedures are established standards; however, policies for direct administration of the investigational medical product to the study participant in a trial are lacking. Current administration rights were examined through the lens of clinical research practices, regulations, and case studies leading to proposed revisions for local adaptation. The authors suggest a standard operating procedure for investigational product that includes a “time out” checklist to ensure improved quality study performance and safety for clinical trial participants. This new standard operating procedure considers evolved quality practices suggested in the new “Good Clinical Practice” guidelines, ICH E6 (R3). With safety and quality at the forefront, this newly proposed SOP has been developed for implementation at the local site. Future research is encouraged.

## Background

**Figure 1.**
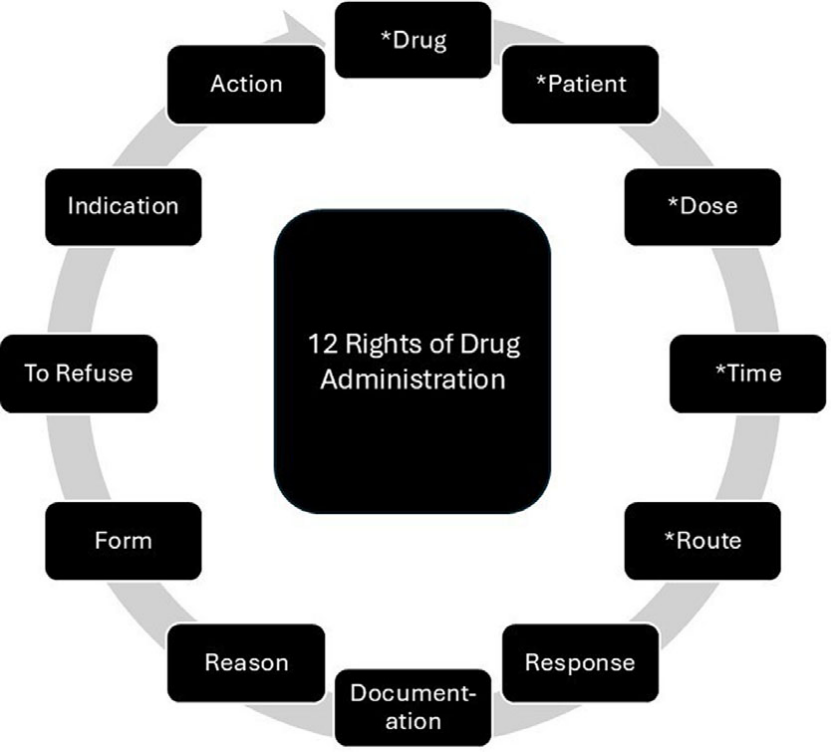
12 rights of medication administration [2]. Note: * = Original 5 rights.

In the clinical care setting, there is an established standard for medication administration that follows a traditional framework called “The Rights of Medication Administration.” While medication errors are the most common and preventable cause of patient injury [1], the five medication administration rights have been globally accepted in clinical practice to reduce medication errors and ensure patient care quality and safety (right patient, right drug, right dose, right route, and right time) [2,3]. This framework has been integrated into daily clinical practice, specifically used by licensed clinical practitioners permitted to administer medications. In 2021, the American Nurses Association released an issue brief expanding the 5 rights of medication administration framework to 8 rights, adding right response, right documentation, and right reason [4]. More recently, this framework was further expanded to 12 rights [3,4]. Those additional rights include: right form, right to refuse, right indication, and right action (Figure 1) [3,5,6]. Regulations and standards that guide clinical medication administration practices are influenced by federal and state laws and agencies.

According to the Code of Federal Regulations, investigational drugs are defined as “a new drug or biologic that is used in a clinical investigation [7].” For this paper, we use the term investigational products (IP) expanding the definition from the International Conference on Harmonization (ICH) E6 (R3) to include approved or unapproved drugs, medicines, medicinal projects, vaccines and biological products, and study-related placebos [8]. Some clinical trials are investigating new indications for an already approved medication. In that context, the off-label medication is also considered IP. Clinical trials using IP require IP administration using precise adherence to an Institutional Review Board (IRB) approved and registered study protocol. As in clinical practice, some medications (including IP) may have a list of disallowed concomitant medications to avoid drug-drug interactions. In clinical trials, adherence to instructions on disallowed concomitant medications is essential to avoid confounding study results and participant safety. Post administration assessments track immediate and other adverse drug reactions to ensure participant safety and reporting of specific study endpoints. All standard of care medication products and study IPs have labels that specify dose, frequency, and length of treatment as well as defined storage requirements as spelled out by the product label or package insert [9]. For clinical trials, the Investigator Brochure, IP label, pharmacy manual, and/or protocol manual of procedures guide the administration, safety considerations, and accountability requirements of IPs.

In hospital settings, the primary administration safeguards for the inpatient and clinic setting are barcode scanning (patients’ identification bracelet and medication) leading to digital documentation and quality checks of the rights of administration. The FDA began requiring bar coding for medications in 2004 including the drug’s unique 11-digit National Drug Code (NDC) to be included [10]. Using barcodes has improved the safety of medication administration in hospitals [11]. Barcodes have been integrated into clinical trial supply chain management and some vendors provide this process for IP [17]. Bar codes can be useful in clinical trial dose administration but are subject to 21 CFR Part 11 requirements, and coding issues for non-approved IPs may exist [12,13]. The FDA has not differentiated the use of bar-coding guidance for IP used in clinical trials from marketed products administered in clinical practice and, at this juncture, is not widely used for IP in clinical trials, although this is emerging [14].

Another safety feature for drug administration includes the use of “time outs.” Time outs have been widely used in the surgical setting before the initiation of surgery and has been incorporated in The Joint Commission processes [15]. The time-out practice has also been adopted as a best practice in the administration of chemotherapeutic agents in oncology [16]. Outside of oncology, a time-out process is lacking in the literature for administration of IP in clinical trials.

## Methods

Recognizing a potential gap in procedures for administration of IP, we initially queried the local investigational pharmacist about the existence of clinical trial IP administration guidelines. None were reported. We then queried approximately 30 clinical research nurses and clinical research professionals across 5 institutions to solicit copies of standard operating procedures for the administration of IPs. All stated that they had standard operating procedures (SOPs) for storage and documenting IP accountability, but none had an SOP for ensuring quality checks for rights of IP administration. We conducted a literature review, for IP administration rights. Our search did reveal the evolution of medication rights for administration of medications (background); however, no articles were found on rights of administration for IPs using the search terms: (“rights of administration of investigational drugs,” “drug administration mistakes in clinical trials,” “procedures for clinical trial medication administration”). We also searched for evidence of bar-coding in clinical trials revealing several supply chain vendors for labels in the gray literature only; however, we could not find specific recommendations for IP bar codes or their use in published literature.

### Case studies

Below in Tables 1 and 2, we share two de-identified case studies illustrating IP administration errors for the purpose of identifying risk issues, corrective and preventive actions (CAPA) and future training topics.

**Table 1.** Case study 1

***Case:*** *A 35-year-old male patient with fungal meningitis was admitted to the research unit to participate in a randomized, double-blind study comparing an experimental IV antifungal study drug, with or without an approved oral antifungal study drug or oral placebo. In the research unit, the patient/ study participant’s chart was flagged with information on the study. In the initial 14-day induction stage of the study, the consented study participant received the IV antifungal drug or IV study drug placebo plus the oral antifungal study drug or oral study drug placebo at the designated doses and dosing schedule per study protocol. On Day 4 of the study, new residents came on service. When rounding on the infectious disease service, the new resident rounded on the study participant in the clinical trial unit. Upon seeing the patient had HIV-related fungal meningitis, he ordered the commercial form of the oral antifungal study drug. The unit nurse obtained and administered the prescribed antifungal drug, despite the patient being on antifungal clinical trial medications. Later that day the clinical research nurse coordinator checked on the patient, noticing the potential overdosage with the commercial oral antifungal drug. An incident report was submitted, and additional safety labs were drawn, given the known liver side effects of the oral drug. The sponsor and Principal Investigator (PI) determined there was no need to unblind the study participant. A protocol violation was documented and submitted to the IRB and sponsor. Additional training was given to the research unit staff and residents assigned to round on that unit. An alert system was put in place to notify the Study RN of any new orders before implementation.* ***What went wrong?*** *Research managers were provided an in service on the protocol and documentation was included in the patient record, included a signed IRB approved consent form; however, some of the research unit nurses were off (maternity leave, vacation) and float pool RNs were assigned to the unit during several shifts [Training gap].* *Drug orders for the study were documented in the participant’s medication record using generic study drug names. The new resident did not recognize the patient was on a clinical trial and did not call the study PI or nurse. [Training gap, Process gap]* *Resident placed a drug order using trademark drug name, which was not recognized by the unit secretary or pharmacy as counter to the study protocol. [Quality process gap, protocol violation, medication error, patient safety issue]* *Pharmacy filled the trade name of the antifungal drug and sent it to the unit to be dispensed, and the unit RN administered to the patient participant [Medication Error, double dosed, potential safety issue, protocol violation]*

**Table 2.** Case study # 2

***Case:*** *A pediatric patient on a cancer study was sent for imaging, per the clinical trial protocol. The study required several study IPs in addition to a non-investigational medication for symptom control. Imaging required an intravenous IP isotope (not investigational, but for the purpose of the study, the isotope was provided in the study).* *During the imaging, the clinical research nurse coordinator recognized that the radiation tech had administered stock isotope instead of the IP isotope. Luckily it was the same concentration, but eCRF documentation posed an issue since not from the study IP. A hospital incident report, protocol deviation, and note to file were filed.* ***What went wrong?*** *Radiation tech did not realize that it was important to use the intravenous IP isotope since it was the same compound and dose. [training gap, quality process gap]* *Isotope infusion was given before the clinical research nurse coordinator was present [quality process gap, protocol violation, patient safety issue, and supply chain documentation issue).*

Given the gap in the literature and lack of SOPs in IP administration at our institutions, we developed an SOP that includes 12 rights of IP administration and an optional “time out” mechanism for point-of-care IP administration in clinical settings, especially in early phase trials. The new ICH E6 (R3), section 3.10 stresses the importance of quality management whereby investigational sponsors and site PIs are responsible for designing studies with a “quality by design” lens that includes risk assessment and risk mitigation from study design through closeout [8]. Medication errors in clinical trials should be reported as incident reports and in some cases as protocol violations when it relates to a clinical trial. To close the quality loop, an analysis and classification of “what went wrong” should be incorporated with a CAPA process.

When an IP is dispensed from the pharmacy, it is the PI’s responsibility to ensure proper handling, dispensation, and administration of the IP [7,9]. Those delegated by the PI to perform IP-related tasks hold significant responsibility to ensure that both protocol and all clinical research regulations are adhered to with utmost accuracy and precision. In outpatient settings, especially outside of the academic medical center setting, a research pharmacy may not be dispensing IP. Many times, study coordinators in private outpatient settings are managing IP accountability, dispensing and administration, under the supervision of the PI. Management of IP is more complex than supply chain management and associated accountability. In the case of IP administration, it also includes assessments, compliance calculations, as well as participant and caregiver IP administration education and adverse event tracking to ensure quality practice and safety. During clinical trial closeout, ICH guidelines requires a final review of IP accountability logs and sponsor approval for IP disposal/destruction [8]. Moreover, FDA regulation 21 CFR 312.62(c) states that an investigator must retain clinical research records for at least 2 years following the date of marketing application approval or even if no application is filed [7].

## Creating a checklist

The authors propose a revised list of IP administration rights for clinical research practice to provide increased safety measures for clinical trial participants to be incorporated into an SOP checklist. These newly proposed rights focus on both regulation and safety. Though the essence of the traditional rights remains, this new set of administration rights cater to the unique nature of clinical research practice.

### Right patient and right drug

The terminology of two of the traditional rights: “right patient” and “right drug,” do not directly translate to terminology commonly utilized in clinical research practice. In clinical research, patients are referred to as study participants. Upon enrollment in a clinical trial, each study participant receives a study participant identification number that is unique to not only that study participant but also to the clinical trial in which they have enrolled. This is not the same as the hospitalized patient medical record number. The assigned study participant identification number has been added as a third required identifier. In a clinical trial the IP may be a drug, biologic, genetic material, or isotope [4]. Moreover, in clinical trials, IPs will use a generic or sponsor coded name “XYZabc” if not already FDA marketing approved for the indication. Randomized, double-blind trials use specific terminology for the IP. The label may state “XYZabc Study Drug” and if double blind, only the pharmacy would have the unblinding records for that study participant and dose. Just as with clinical medications, IPs have accurate pharmacy labels associated with a prescribing investigator’s order. However, IPs also include study specific dispensation identification numbers on their labels (i.e., kit or lot numbers) and the phrase “for investigational use only.” These numbers are exceptionally important in ensuring that the correct IP is dispensed to the correct study participant. Therefore, the dispensation identification number is an essential criterion for the IP verification process. Other aspects of IPs that must be considered include expiration date/time verification and IP storage considerations. If the IP requires reconstitution, reconstitution requirements must be followed and a date/time for the reconstituted-product expiration will also apply. Additionally, IPs must be kept in protocol specified storage conditions. Temperature (ambient, refrigerated, frozen) and light exposure will be explicitly dictated. Storage conditions must be thoroughly and adequately logged. Any excursions from the specified conditions must be reported and may render the IP unacceptable for administration and be considered an excursion protocol violation.

### Right dose, route, and timing

The terminology for the last three of the traditional rights: “right dose,” “right route,” and “right time” remain unchanged; however, randomization, blinding, titration requirements, and recordkeeping processes may be important considerations. For instance, dosing may not follow the traditional timing of dispensing a drug but may have very specific time-related protocol requirements for associated study procedures with dose administration [3].

In clinical trials, “right dose*”* is dictated by the clinical trial protocol and randomization process. Further, there are times where the dose may include a titration plan for efficacy and/or safety. As such, the IP label must be compared to both the IP physician order and the study protocol. The criteria for “right route” require verification of the administration route indicated on both the medication label and the clinical prescription. Just as with “right dose,” the administration route for an IP is dictated by the trial protocol and randomization schema (if a controlled randomized study). Therefore, the IP label must be compared to both the prescription and the trial protocol for verification. Moreover, in the case of a blinded study, there are policies defined in the protocol to ensure blinding and if necessary, the parameters for requesting and allowing unblinding [17]. Very few trials allow unblinding unless encountered with life and death situations [17].

Lastly, “right time” is traditionally defined and satisfied through the administration of a medication at the date, time, and frequency prescribed. In clinical practice, administering a medication at the “right time” merely requires verifying the time indicated on the prescribed order and ensuring that the medication is administered at the prescribed time of day, frequency, and/or interval and instructions on how to handle missed doses. Administering an IP in a clinical trial may be more complicated and precise. Not only must date and time of day be considered but there are other critical timepoints that may be required in clinical trials. For instance, the trial protocol dictates what, if any, study-related procedures must be completed within a particular study visit prior to or after IP dose administration. These may include imaging, questionnaires, clinical tests, surgeries/procedures, and laboratory testing. Laboratory testing may include safety labs, urine pregnancy tests, biomarkers, biobanking, autoantibodies, pharmacodynamic, and/or pharmacokinetic samples. Some early phase studies may also include pharmacokinetic and pharmacodynamic sampling performed at specific timepoints throughout a given study visit.

### Additional rights

In clinical practice, *the* “right reason” operates as a safety measure to verify the reason for a given medication being prescribed and administered to a particular patient [6]. “Right reason” in relation to clinical research practices involves ensuring that the study participant has met all inclusion and exclusion criteria for clinical trial enrollment. In our proposed SOP, we are substituting the term “right eligibility” for “right reason.” Additionally, “right documentation” involves ensuring that an IP order and administration specific to the clinical trial has been accurately documented. IP accountability and compliance requirements include tracking dispensation, return, and self-administration accuracy. “Right documentation” may vary based on the specific clinical trial and having the right documentation on file for IP administration is critical. In outpatient settings, patients can document self, or caregiver assisted administration in a study drug diary. “Right response” includes a participant’s response to a given IP. Unlike FDA approved medications, the potential side effects and adverse reactions for IP may not be comprehensively known. This right also includes an assessment of the effectiveness of a medication in addressing the intended medical condition [5]. For instance, some protocol objectives and endpoints may include timed outcome assessments, such as “temperature before and at 12-hours after initial dose of study medication.”

We incorporated *“*right form*”* and *“right to refuse”* in sections related to “right documentation” and “right informed consent.” Clinical trial protocols have a sponsor protocol number and after rigorous review, IRB also designate an IRB review and approval number [11]. Both the sponsor provided protocol ID number and the IRB number serve as unique study tracking numbers that serve as a verification tools which study staff can use to ensure that any IP to be administered is for the correct trial. As such, we include “right clinical study*” as* one of the essential rights for IPs.

Table 3 includes the rights, definitions, and checklist criteria for the proposed IP rights of administration. Table 4 incorporates the checklist into a proposed SOP.

**Table 3.** Investigational medicinal product administration rights checklist

Rights of IP Administration	Definitions	IP Administration Checklist Criteria
**Clinical Study Protocol/Version**	Sponsor’s protocol ID/number and/or IRB Number	Compare Sponsor’s protocol ID/number and/or IRB number on order with IP label Verify using the most recent IRB approved version of the protocol.
**Study Participant** **[Patient]**	Two personal identifiers One study participant identifier	Compare at least 2 personal identifiers (i.e., name, DOB, MRN) and 1 study participant identifier (study participant’s identification number) on order with IP label.
**Eligibility**	Inclusion/Exclusion Criteria	Verify that all Inclusion and Exclusion criteria have been met to confirm clinical trial eligibility (for initial IP administration). If required, re-evaluate eligibility during staged decision points (e.g., titrating to a new dose level)
**Study Informed Consent**	Active IRB approved study consent form signed by the participant, legal guardian and/or LAR, if applicable. Active IRB approved study assent form signed by the participant, if applicable.	Verify that the study participant, legal guardian, or LAR has signed an active, IRB approved clinical study consent form, as appropriate. Verify that the study participant has signed an active, IRB approved clinical study assent form, as appropriate.
**Investigational Medicinal Product** **[Drug]**	Protocol specific investigational medicinal product name. Study visit-specific dispensation identification number(s). Expiration date, if available Beyond use date/time, if applicable ***IP Randomization and Blinding (if applicable)*** Follow randomization procedures. Adhere to blinding per protocol (open-label, single blind, double blind) ***IP Storage*** Store IP in specified conditions (per IB or protocol) Follow and document storage and temperature requirements	Compare the IP medicinal product name and dispensation identification number(s) on the IP label with the order. Check expiration date to verify IP has not expired, if available If applicable, check beyond use date/time (e.g., reconstituted IP) If applicable, verify the correct additives (e.g., reconstitution requirements, specific IV fluids, administration kits). If applicable, verify the correct flush to be used with the IV IP. Follow randomization procedures to ensure proper dose dispensing (IP vs SOC or placebo). If blinded, do not unveil the blind outside of protocol-specific allowances.
**Dose**	Protocol specified dose. Protocol specified dose units. If applicable, protocol specified concentration.	Compare the dose and dose units, and concentration of the IP, if applicable, on the IP label with the order and protocol.
**Route**	Protocol specified route of administration *(oral, subcutaneous, intramuscular, intravenous, etc.)*	Compare administration route on the label with the prescribing investigator order. Administer the study product per route ordered. If applicable, verify the IV IP can be administered via IV lines receiving specific fluids, biologic materials, and/or drugs
**Time**	Protocol specified visit(s) and dosing schedule. Protocol specified time and frequency during each study visit	Administer the IP at the right visit according to the study protocol. Administer the IP at the right time during each visit in relation to labs and/or testing procedures as indicated in the protocol.
**Documentation**	Protocol and institution specified required administration documentation.	IP order completed and signed by the ordering provider for each administration instance. IP administration documentation must be completed for each on-site administered dose. If applicable, IP accountability must be performed and documented (i.e., dispensation and returns, quantity/volume returned, identification of missed doses, IP destruction (if allowed). If applicable, IP compliance must be calculated.
**Response**	Assessment of IP side effects and adverse reactions safety endpoints (AE, ADR, SAEs). Assessment of IP effectiveness (efficacy endpoints; compliance).	Perform and document protocol required side effect and adverse reaction assessments at each study visit Perform and document protocol required IP effectiveness assessments at each study visit (includes IP compliance assessment), if applicable

Note: Abbreviations: AE = adverse event; ADR = adverse drug reaction; SAE = serious adverse event; IP = investigational medical product; DOB = date of birth; MRN = medical record number; IP = Investigational product; IB = Investigational Brochure; IRB = institutional review board; LAR = legally authorized representative; SOC = standard of care.

**Table 4.** Investigational product administration SOP with time-out checklist

**Title: A quality procedure for investigational medicinal product administration to clinical trial participants in the hospital, clinic, or home visit setting.** **SOP #______** **Effective Date:_______** **Revision date(s):______________** **Purpose** This SOP seeks to mitigate IP administration gaps to ensure the safety and care of study participants and to adhere to study protocol requirements for IP administration. **Scope** This SOP is pertinent to hospitalized (inpatient) administration of study IP or study drug administration in a hospital, clinic or a home visit, as required. A separate SOP for outpatient self-administered IP by the participant is: #_______ and is customized for the associated protocol. **Key Performance Indicators**: correct administration of investigational product: Right clinical study protocol, right study participant, right eligibility, right study informed consent, right IP, right dose, right route, right time, right documentation and right response. ii. **Key Risks**: improper administration can cause medical risks to study participants; as protocol violations, may jeopardize study integrity. Medication errors should be promptly reported through an incident report and a protocol violation report. **Definitions/Acronyms** **Investigational Product (IP)**- “A pharmaceutical form of an active ingredient or placebo being tested or used as a reference in a clinical trial, including a product with marketing authorization when used or assembled (formulated or packaged) in a way different from the approved form, or when used for an unapproved indication, or when used to gain further information about an approved use. Investigational products should be considered synonymous with drugs, medicines, medicinal products, vaccines and biological products.” (ICH R6 (R3) Good Clinical Practice, Glossary, page 75). b. Timeout Checklist- “*a standardized process, initiated by a member of a team, involves immediate members (at least one other) to verify at a minimum: correct patient identity, correct site, correct procedure.”* This SOP uses a time out prior to administering study IP. A member of the team documents the time-out query answers, usually digitally. Time out has also been used in the administration of ICU medications and chemotherapy. (The Joint Commission, 2025, National Patient Safety Goals, UP.01.03.01, EP 1-5; Tainter et al, 2018; Kalo et al, 2019). **Procedures** Establish team (at least 2, one RN) for administration of the IP. Obtain the IP and other equipment needed for dosing. Double check dosing materials against checklist (below) before entering patient room Enter room and perform introductions. Start a time out and go through checklist orally (using rolling laptop or iPad). (Note: the checklist may be integrated into a digital database, accessible on a mobile device. Screenshots can be integrated into training materials. *(Insert checklist).* Administer IP to study participant. Check for response, as indicated Document the process. ***SOP References***

### Planned pilot study

Before implementing this SOP more widely in the institution as policy, we will pilot it in two pediatric clinical research areas: the Schubert Research and Vaccine Research clinics. A REDCap database will incorporate checklist elements and be tested before use in the study. We will be tracking ease of use of digital checklist during the time-out, logistical feasibility of dosing hospitalized or outpatient clinic participants using the time-out feature, total minutes of the SOP execution (pre-time-out and during time-out). During the study period, we will capture medication errors that occurred, near misses caught prior to and during the time-out sequence. Study personnel and staff that participated in the study will complete brief mixed methods surveys to collect information about errors, usability of the process and digital materials.

After IRB approval, we will conduct webinar in-services on the study IP administration procedures, surveys and equipment for the study units and study staff involved in specific studies. At the end of the six-month study period, data will be analyzed. Modification of the checklist SOP will be incorporated, based on feedback. Pilot study results will be disseminated.

## Conclusion

Clinical research continues to grow in complexity. Errors in IP administration jeopardize patient safety and the integrity of the protocol. Many errors can be avoided when quality checks are put in place. Moreover, with the recent adoption of a revised GCP guideline (ICH E6 (R3), sponsors, investigators, institutions, and site study teams are challenged to incorporate quality by design and risk-based approaches to mitigate errors and harm. As part of a quality improvement effort to reduce medication errors in clinical research, we re-examined the existing rights of medication administration and propose IP rights of administration and an associated SOP that includes a time-out checklist for IP administration. This SOP enhances other IP-related SOPs that are common at clinical research sites related to study products such as “Managing Study Supplies” and “Drug Storage and Accountability.” Similar SOPs should be developed to address the use of investigational devices which may have more complex quality checks and procedures. Future quality improvement studies should be conducted using this or other tools that may improve quality, efficiency, and safety of conducting clinical trials.
